# Recruitment of Yoruba families from Nigeria for genetic research: experience from a multisite keloid study

**DOI:** 10.1186/1472-6939-15-65

**Published:** 2014-09-02

**Authors:** Peter B Olaitan, Victoria Odesina, Samuel Ademola, Solomon O Fadiora, Odunayo M Oluwatosin, Ernst J Reichenberger

**Affiliations:** 1 Department of Surgery, Ladoke Akintola University of Technology, College of Health Sciences, Osogbo, Nigeria; 2 Department of Medicine, University of Connecticut Health Center, Farmington, CT 06030, USA; 3 Department of Surgery, Division of Plastic Surgery, University of Ibadan, Ibadan, Nigeria; 4 Department of Reconstructive Sciences, Center for Regenerative Medicine and Developmental Biology, University of Connecticut Health Center, 263 Farmington Avenue, Farmington, CT 06030-3705, USA

**Keywords:** Keloid, Recruitment, Genetics, Families, Yoruba, Nigeria, Low resource settings

## Abstract

**Background:**

More involvement of sub-Saharan African countries in biomedical studies, specifically in genetic research, is needed to advance individualized medicine that will benefit non-European populations. Missing infrastructure, cultural and religious beliefs as well as lack of understanding of research benefits can pose a challenge to recruitment. Here we describe recruitment efforts for a large genetic study requiring three-generation pedigrees within the Yoruba homelands of Nigeria. The aim of the study was to identify genes responsible for keloids, a wound healing disorder. We also discuss ethical and logistical considerations that we encountered in preparation for this research endeavor.

**Methods:**

Protocols for this bi-national intercultural study were approved by the Institutional Review Board (IRB) in the US and the ethics committees of the Nigerian institutions for consideration of cultural differences. Principles of community based participatory research were employed throughout the recruitment process. Keloid patients (patient advisors), community leaders, kings/chiefs and medical directors were engaged to assist the research teams with recruitment strategies. Community meetings, church forums, and media outlets (study flyers, radio and TV announcements) were utilized to promote the study in Nigeria. Recruitment of research participants was conducted by trained staff from the local communities. Pedigree structures were re-analyzed on a regular basis as new family members were recruited and recruitment challenges were documented.

**Results:**

Total recruitment surpassed 4200 study participants over a 7-year period including 79 families with complete three-generation pedigrees. In 9 families more than 20 family members participated, however, in 5 of these families, we encountered issues with pedigree structure as members from different branches presented inconsistent family histories. These issues were due to the traditional open family structure amongst the Yoruba and by beliefs in voodoo or in juju. In addition, family members living in other parts of the country or abroad complicated timely and complete family recruitment.

**Conclusions:**

Organizational, logistics and ethics challenges can be overcome by additional administrative efforts, good communication, community involvement and education of staff members. However, recruitment challenges due to infrastructural shortcomings or cultural and religious beliefs can lead to significant delays, which may negatively affect study time lines and expectations of funding agencies.

## Background

While most research on common or rare disorders has in the past been conducted in European and East Asian populations, there is increasing interest in conducting such studies in sub-Saharan African populations. There is a growing recognition that health disparity research will be improved by including populations with genetically diverse background. This means we need to include more sub-Saharan Africans in genetic research for common and rare-disorders, especially as this region is known to harbor the greatest amount of genetic variation within our species [1].

Within the United States, minority recruitment for genetic/family studies has been recognized as crucial to understand genetic variation and to enable generalization of research findings. Pharmacogenomic studies, for example, investigate the efficacy of pharmacologic agents in populations with certain genetic characteristics [2-4]. Genetic research in African populations is needed to study the genetic predisposition of Africans to certain diseases. Currently there are projects under way to investigate proposed rheumatoid arthritis loci in African populations [5], to investigate susceptibility of Nigerians to risk loci for chronic kidney disease [6] or to study the effect of certain promoter variants for modulation of serologic autoimmunity to SLE [7].

The Yoruba ethnic group of Nigeria has been sampled in previous genetic studies. The Yorubas were part of the international HapMap studies that led to the sequencing of the Human Genome [8] and to create a map of human genome sequence variations [9]. Samples from the Yoruba ethnic group were also included to collectively identify >3 million common DNA variants, primarily SNPs [10]. SNP data and their linkage disequilibrium patterns provide basic information for genome-wide association studies in African or admixed populations [8,10-13].

However, to our knowledge there are no genetic studies that use the Nigerian or Yoruba population as their primary target. Our study is in the process of identifying the genetic basis for keloid formation, a wound healing disorder, with the Yoruba ethnic group as the primary target population.

**Figure 1:**
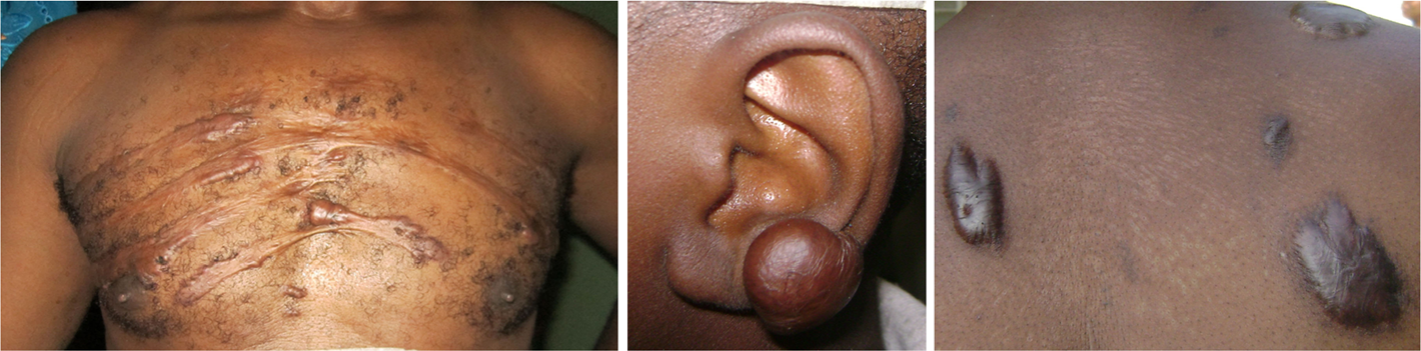
Patients with typical keloid scars on sternum, back and earlobe.

Keloids are scars that grow over the margin of the original injury to the dermis (Figure 1). This often disfiguring, painful, and stigmatizing scar can occur after a minor cut or trauma to healthy skin. Keloids occur mostly in dark pigmented populations [14,15] including the Yoruba people of Nigeria. There is no good and efficient treatment for keloids [16,17]. It has been generally accepted that there is a genetic basis for keloids [18-21]. However, the mode of inheritance is still unsettled. Some studies suggest that the keloid inheritance pattern is autosomal dominant [21,22] while a study of 34 Yoruba family pedigrees (from Nigeria) supported autosomal recessive inheritance [23].

The Yoruba awareness of keloids predates the first publication on keloids in Western literature [24,25]. According to Omo-Dare, Yorubas for long have referred to keloids in their art and literature [26]. Indeed, one of the 140 chapters in the Ifa literary corpus is dedicated to keloids; with artifacts/sculptures and folklore reflecting keloids. The Yorubas, with their local customs of facial markings and earlobe perforations, recorded their familiarity with keloids 10 centuries before Alibert and Retz [27].

For modern-day genetic research, it is often necessary to recruit thousands of participants, especially for association studies. In the instance of case-control studies, only a single affected participant or a single unaffected control individual per family is recruited. Other strategies involve the recruitment of trios (father, mother, and affected offspring) or sib-pairs. Recruitment for those studies may be relatively straight-forward, although not trivial. For linkage studies, however, large families must be identified where the disorder of interest is transmitted. Challenges arise when many informative family members need to be recruited to give enough power to a pedigree for independent identification of a disease gene locus. Developing strategies to present the research to potential study participants in a manner that enhances voluntary participation of family members from all generations becomes essential, and can be a challenge.

Suggestions to enhance recruitment for genetic/family studies with emphasis on minority recruitment in the US or recruitment in Africa have been addressed in the literature [28-31]. However, literature on recruitment strategies or recruitment experience for genetic/family studies in the Yoruba population is limited. This article documents our recruitment experience for a genetic linkage study in the Yoruba population in Nigeria and describes the challenges we faced as well as methods used to reduce these challenges.

### Setting: Nigeria

Nigeria is a country in West Africa with an estimated population of 170,000,000 (census 2010), which is composed of 250 ethnic groups including Hausa and Fulani (29%), Yoruba (21%), Ibo (18%), Ijaw (10%), Kanuri (4%), Ibibio (3.5%), and Tiv (2.5%). Nigeria was a port for the slave trade and obtained its independence in 1960 from Great Britain. The official language is English but the three major local languages are Hausa, Yoruba, and Ibo, along with more than 200 dialects. The Yoruba homelands are in the western part of Nigeria, the Igbo reside in the east, while the Hausa and Fulani live in the north.

### Lessons learned

This study was carried out with careful consideration of cultural issues in the host country. We followed recommendations to enhance recruitment in such low resource-limited settings [29,31]. Here we share our experience on several issues that impacted our recruitment for consideration in future studies.

## Methods

### Recruitment sites

Participant recruitment was carried out in Ibadan (Oyo State) and Osogbo (Osun State) as well as in surrounding towns and villages. Ibadan is the capital of Oyo state with a population of 1.3 million, the third-largest metropolitan area in Nigeria after Lagos and Kano. Ibadan is located in south western Nigeria with the principal inhabitants being ethnic Yoruba. There are several smaller towns and villages around Ibadan where recruitment of study participants took place including Ogbomoso, Egbeda, Oyo, Iseyin and others. Osogbo, the second recruitment site for this study is the capital of Osun State with a population of approximately 160,000 (by 2006 census). Most of the inhabitants are Yorubas. Recruitment around Osogbo extended to surrounding towns, including Ile-Ife, Modakeke, Ikirun, Ilesa, Ede, and Ikire, among others. The population structure and culture in Oyo and Osun States are very similar.

### Study preparations and interaction between US and Nigerian research groups

Potential study sites and plastic surgeons who were interested in a genetic study were identified through personal contact with a health care professional in Ibadan. The US researchers worked with the local researchers from the inception of this project. Ethics approval from an IRB in the US and ethics committees in Nigeria was obtained for the initial pilot study prior to the US researchers’ first visit to Nigeria. During the first visit to Osogbo, which was paid for by institutional funds and by the University of Connecticut Health Center (UCHC) General Clinical Research Center (GCRC), the US scientists met with Nigerian plastic and general surgeons and chief medical directors of different hospitals as well as with provosts of colleges of health sciences to describe the goals and objectives of the research project. Discussions followed on the prevalence of keloids in their clinics and the surrounding communities, as well as on how to engage the community leaders and how to dispel fears and concerns related to research amongst the keloid patient population. Plans were developed on how to address cultural beliefs, and which methods would be most appropriate to promote the research study. Also discussed was the feasibility of hiring staff through local hospitals, research and ethics training for staff, plans for transporting samples from surrounding towns, data collection and the transfer of samples to the US. Additional activities included FWA registration of the Nigerian institution in Osogbo and human subjects training (CITI) for those investigators who did not already have that certification. As a team, we outlined a feasible roadmap for the pilot study with the goal of obtaining NIH funding (R01) for large-scale recruitment.

Communication between the Nigerian teams and the research group from the US involved emails and telephone contacts between regular visits to Nigeria. Consenting and privacy (HIPAA) documents were reconciled regularly to be acceptable to the ethics committees and IRB on both sides. As NIH funding was obtained, subcontracts between institutions were developed and an additional recruitment site in Ibadan was instituted. Investigators reviewed recruitment processes collaboratively, planned and implemented recruitment in surrounding towns and villages and updated staff training to the Nigerian research teams during regular visits throughout the recruitment period. While in Nigeria, the US team took part in recruitment efforts, meetings with community leaders, educational forums and media presentations.

### Study design

The primary goal of this cohort study was to recruit families where keloids are inherited in an effort to identify the genetic basis for keloid scar formation. The focus of recruitment was on large multi-generation families. Keloids were assumed inherited when several members of a family were affected. Probands who did not report any additional affected family member were classified as sporadic or singular cases. Data discussed here were obtained between August 2005 and February 2013.

Ethics approval was obtained from the Institutional Review Board of the University of Connecticut (IRB#03-007) and the ethics committees of the Ladoke Akintola University of Technology (LAUTECH), College of Health Sciences, Osogbo and the College of Medicine, University of Ibadan/University College Hospital (UI/UCH), Ibadan. All oversight committees had obtained Federal Wide Assurance (FWA). Recruitment of study participants was performed by trained recruitment teams under the supervision of plastic surgeons at LAUTECH and the (UI/UCH). Recruitment was supported by NIH-funded subcontracts with both institutions. Each recruitment team consisted of trained nurses, research assistants and two surgeons to direct the recruitment efforts as co-investigators and to diagnose participants. Training of staff included human subjects training as well as training to differentiate between keloids and other wound healing disorders (e.g., hypertrophic scars). All team members and surgeons were Yorubas who lived in the community and were employed at the respective teaching hospitals. The US research team included a Yoruba nurse research associate who was the professional and cultural liaison between the research teams from the US and Nigeria and the Nigerian community. The American research team travelled to Nigeria 1-2 times a year to provide support and technical assistance to the Nigerian teams.

### Recruitment strategies

Probands were recruited either in the hospitals at LAUTECH in Osogbo and UCH in Ibadan or in the community. Outside the hospital settings, participants were recruited in private and public clinics, private homes or by invitation in churches and mosques.

#### Patient advisors

We utilized a modified community-based participatory research approach to engage keloid patients and the community at large. A number of keloid patients who were recruited in the early phases of the study served as patient advisors. The research teams educated them about the goals of the project and the involvement of research participants. Patient advisors assisted by passing information about the project to individuals with keloids in their communities, and instructed them to contact the recruitment team if they were interested in participating. While patient advisors were generally able to answer many questions of participants, they did not participate in the actual recruitment.

#### Community leaders

To initiate recruitment in a new town, the groups visited the king or his chiefs and ward leaders, who hold important leadership roles within the community, to explain the purpose of the research and the need for cooperation of the population. At those meetings the research team could answer questions from the leaders and/or the community. Posters and handbills (flyers) were distributed during the visits.

#### Health directors

Medical and nursing directors of local health centers and private hospitals were approached with the request to identify study participants which would then be recruited by the Nigerian research staff. Posters and handbills with pictures of keloids were made available in the clinics to introduce the research project to patients.

#### Churches/mosques

We solicited the assistance of pastors and imams to share the research study with their congregations. The religious leaders gave us permission to explain the study during a special announcement period. Research teams stayed after the service to answer questions and set up appointments for recruitment.

#### Advertisement

Handbills and posters were distributed in the communities, specifically in market places, private and public health clinics, schools, churches, pharmacies and clinical laboratories. The posters and handbills in English and Yoruba language contained information about the study, contact information and stipend.

#### Media

The research team made radio and TV presentations in English and Yoruba language describing the purpose of the study, eligibility criteria and contact information. In addition, radio and television announcements were run to advertise the study and to dispel some myths about keloids.

#### Affected/enrolled participants with keloids

Often times, enrolled participants were asked to spread word about upcoming recruitment visits in their community. As a result, additional keloid patients usually came to the recruitment site to join in the study.

### Participant recruitment

Recruitment and enrollment of participants took place in the plastic surgery clinics in Ibadan or Osogbo hospitals and in the community (participant’s home, work place and other chosen site). Diagnosis of keloids was confirmed by the clinicians heading the recruitment teams.

The consenting process involved explaining the study to potential participants. Prospective participants were given copies of the consent form and related documents to read or have them read to them.Participants were given sufficient time for review and to ask questions. They had the option to participate the same day or at a later day if they would like to discuss further with their family. Usually, married women liked to discuss with their husbands before agreeing to participate. After consent was obtained, the recruiter and the research participant filled a questionnaire to document the participant’s personal information, keloid history and past medical history. Minors between ages 7 and 12 signed assent forms and in addition, the recruiter obtained parental consent. Older minors signed the consent forms together with a parent. Keloid site(s) and sizes of lesions were documented in a questionnaire (body map) and photographs of keloids and other scars were taken. Participants consented to have images of their keloids (Figure 1) used in scientific articles. Research assistants constructed pedigrees when probands reported other affected family members.

Probands were asked to inform other family members about the study so they could contact the research team if interested. Family recruitment often took place in their homes which required the research team to travel to their towns or villages. We also recruited large cohorts of patients with keloids without family history (or with no other family members available for the study) and control individuals without keloids. Eligibility criteria for participation as unaffected control individuals were the absence of keloids or suspicious scars and that no member of their families had keloids, suspicious scars or skin diseases. Only one control individual per unaffected family was recruited. Recruitment settings for controls were the same as for the keloid case recruitment. Contact information of all participants was collected in case clarifications were required during the course of the study. Participants were given a sum of approximately $10 (USD) (Naira 1,500.00) as compensation for transportation and inconvenience. Venous blood samples (Vacutainer, Becton Dickinson, USA) or saliva samples (OraGene saliva kits; DNA Genotek, Ontario, Canada) were collected and transported to the US for processing at the University of Connecticut Health Center. Each participant was given a unique identification number.

### Analysis

Pedigrees were entered into Progeny pedigree drawing software (Progeny Software, LLC, Indianapolis, IN, USA) and were updated whenever new information about a family was obtained. Statistical analyses were performed in Excel and SPSS. Challenges observed in the process of recruitment and issues that could affect genotype analysis were documented.

## Results

### Administrative challenges

Before the actual study could commence a number of administrative steps were necessary to establish agreements between institutions, to train research staff and to ensure that ethics approvals were in place so that the project would accord with the latest standards of human subject protection.

The first recruitment site for our study, LAUTECH Teaching Hospital, Osogbo, Nigeria, was founded in 2000 with limited experience in collaborative research with foreign institutions. The first challenge for establishing a research relationship between an established institution in the United States and a new Nigerian teaching hospital was in regards to human subject protection and finance administration (see the List of administrative challenges section). First the Nigerian site had to register with the United States Department of Health and Human Services to receive a Federal Wide Assurance (FWA) number. Principal Investigators needed to complete human subjects training with the Collaborative Institutional Training Initiative (CITI) before they could be added to any research protocol approved by the University of Connecticut Health Center (UCHC) Institutional Review Board (IRB). Consenting documents had to be crafted to suffice all human subjects protection elements and oversight requirements for UCHC protocols. Problems for the Nigerian ethics committee arose when paragraphs were included that are only relevant to the US but may be confusing to participants in Nigeria. Examples of where study participants can get confused are references to:

a) Health Insurance Privacy Portability Act (HIPAA) and health insurance providers, because health insurance is unavailable to most Nigerians;

b) Requirements of a specific US State, which do not apply to Nigeria;

c) US oversight committees that may gain access to consenting materials (Protected Health Information (PHI) in case of project audits.

Consents and HIPAA forms need to be carefully crafted to consider the environment and culture of research participants [31]. Recruitment materials needed to be approved by ethics committees/IRBs of both institutions. Training of all research staff in ethics and research compliance was needed before any approval could occur. Training was performed by the US staff during extended visits.

#### List of administrative challenges

● Inexperience of investigators and institutions in international collaborations

● Raising funds for pilot study to demonstrate feasibility of study to funding agencies

● Registration of Nigerian recruitment sites for Federal Wide Assurance (FWA)

● Adapting wording of consent/HIPAA forms to local requirements

● Reconciling IRB requirements of US and Nigerian institutions

● Human subjects training of new recruitment teams

● Implementation of subcontracts with different expectations of financial administrations

● Maintaining ongoing communication between teams to optimize recruitment and for trouble shooting

● Funds transfer due to differences in banking systems

Significant start-up funds were needed to initiate this collaboration as funding agencies (e.g., NIH) will not allow spending funds abroad until all compliance issues are fulfilled and the foreign site is registered with the funding agency. However, in order to start such a genetic collaboration from scratch, travel costs, resources for salaries in the host country as well as computers, internet connection, cameras for documentation, recruitment documents and operating funds had to be funded in advance. Most institutions in low resource countries cannot afford to finance salary expenses upfront and be reimbursed later. Whenever banking systems are different, even the transfer of funds in itself can pose an administrative challenge. Since Principal Investigators at UCHC and LAUTECH had no prior experience with the administrative and regulatory challenges of such a collaborative project and because such a large international genetic study was new to administrations at both sides, our project suffered significant delays.

The University of Ibadan, which was later added as a second study site, had experience with international collaborations as it had participated in previous research activities including the HapMap project. As a result of the experience gained with establishing the first recruitment site at Osogbo, it was easier, although not trivial, to add the University of Ibadan as the second recruitment site.

### Recruitment challenges

There were several other challenges to successful recruitment (see List of recruitment challenges section). In most families there were some members who lived far away, often located throughout Nigeria and even abroad. Those who lived within the country but in other states did not return to their home towns every year, which prevented timely and complete recruitment of many large families.

#### List of recruitment challenges

● Obtaining referrals from clinics and community centers without payment

● Unreliable transportation system and road infrastructure

● Participants are less motivated to participate in research without direct (health) benefits

● Participants reside in remote locations

● Participants lack funds to maintain mobile phones

● Participants do not show for appointments

● Difficulty to understand research project and purpose

● Inaccuracies in family and clinical histories

● Lack of birth records for older adults

● Open family system which does not distinguish between biological siblings and adopted or socially related siblings

● Polygamy with families living far apart

● Hesitation to donate sample because of beliefs in “voodoo” and “juju”

● Paternity issues

Furthermore, even though most people had mobile phones, communication was problematic at times as most probands did not have contact to all of the family members. Frequently, the research group had to travel long distances for recruitment or to confirm pedigree structures, especially in polygamous pedigrees where members from one branch of the family had insufficient knowledge of other parts of the family. On the other hand, we found that children from one wife sometimes lived with families of other wives and were fully accepted as children of the family. Information about “brothers” or “sisters” had to be confirmed by several sources since in many cases, families did not distinguish between “sibling” or “half-sibling”. Also, in this open family system, other relatives living with families were often reported as immediate family members and were therefore wrongly recruited.

For travel within the state, the recruitment teams had to rely on public transportation. Public transportation was plagued by unreliable connections; hence it was frequently time consuming to commute to recruitment sites. Poor road conditions made travel even more challenging, thus reducing staff time for actual recruitment. Many of the participants (older generations) did not have formal education and found it difficult to understand the research concept with no immediate benefit for their keloids, which they often viewed as a stigmatizing condition. They were more interested in a study that treats or cures keloids and not particularly eager to participate in a study that will take years before yielding meaningful results. Some individuals only wanted to participate if they could receive free treatment.

We explored access to keloid patients in Ibadan and Osogbo or in neighboring towns through local hospitals, community clinics and private clinics. This approach often failed as clinicians, especially in private clinics, were reluctant or not willing to inform keloid patients about the study for fear that patients could be diverted from them. Some health care providers (including some hospital staff at host institutions) expected payment for patient referrals to the study, commonly referred to as “head money”. We considered payments for referrals as unethical.

To reimburse any clinic for actual time and efforts spent on recruitment, we would have needed a formal contractual agreement. Establishing a formal contractual agreement would have been a major administrative effort, necessitating involvement of the funding agency and institutions managing the funds.

Accurate pedigrees and clinical histories were sometimes difficult to obtain for several reasons. Some participants, especially those without formal education, had no records of their birth dates and hence were guessing their age, which was at times far off when we compared the parental age to that of the children, who did have birth certificates and some level of education. This problem was more common with women who did not attend school and had no need to obtain official birth records. We were therefore aware that we may have received incorrect information regarding age or relationships despite direct questions.

In some instances, we found discrepancies with genotyping results and had to re-visit families for clarification. We had to exclude several individuals in key families for our linkage study because participants could no longer be reached or discrepancies could not be fully resolved. Other issues included friends posing as family members, or that individuals were addressed by different names by different members of the family, which is not uncommon in the Yoruba culture. These cultural idiosyncrasies made it necessary to re-evaluate pedigrees whenever new participants were recruited. For some families it took 5 years to recruit all informative family members who were interested in participating.

There were circumstances where remuneration became a major factor for recruitment. While most study participants were happy with the amount of compensation, some participants tried to negotiate for higher compensation. Some individuals believed they should be paid more because they had more keloids or because they had a longer way to travel to a study site. Others wanted extra payment before they would introduce the research team to other family members. Recruitment teams sometimes had to provide prepaid phone cards to probands who were unable to call other family members because of their financial situation. Occasionally, there were extended delays in communication when family members could not be reached because they lacked funds to purchase phone cards. Some private clinics or probands indicated that they were willing to work with the research team if we let them distribute the study stipend to referred participants. This would have allowed them to split the remuneration after subject enrollment.

The study also faced challenges from religious beliefs and cultural practices. The Yorubas have strong beliefs in voodoo or juju, which vary within religious groups. Some were convinced that their saliva and/or blood would be used for evil rituals. As a result, families withdrew from the study and the recruitment teams had to destroy some samples that were already collected.

We strongly believe that deeply ingrained cultural beliefs also played a role when research teams had difficulties to re-contact study participants. In such circumstances, participants would find excuses or make appointments with the recruiters and not show up rather than frankly telling the research team that they were afraid of voodoo or juju or do not wish to participate for other reasons. This led to further delays in the recruitment progress and sometimes was a dead end for the recruitment of a family that would have been informative for the genetic study.

During sample analysis, we discovered inconsistencies with paternity and false allocations of children from different wives. We also encountered friends or distant relatives posing as close family members in at least 5 out of more than 100 families. Recruitment in 3 large families from the Osogbo group and 10 families from the Ibadan group were delayed significantly because family members relocated to different parts of Nigeria or to foreign countries. Three Osogbo-based families withdrew from the study or could not be recontacted for unknown reasons while 9 families withdrew or could not be re-contacted by the Ibadan recruitment center. The fear of voodoo or juju prohibited the recruitment of a larger number of family members in three families in Osogbo while 5 individuals raised the issue of juju among prospective participants in Ibadan. Most other family members could usually be recruited.

Total recruitment exceeded 4200 study participants over a 7-year period (Table 1). Sample collection in a number of families was incomplete for various reasons, either because family members refused to participate for fear of rituals or for unknown reasons or because of family members living in other parts of the country. Participants who had no family history or where no other family member was available for recruitment were included in a case cohort for a future genome wide association study (GWAS). We were able to recruit approximately 1,900 individuals with keloids during the recruitment period. In preparation for a GWAS, we also recruited more than 2,000 unaffected control individuals. Our initial goal, however, was to recruit mostly large families with inherited keloid susceptibility. We identified and recruited from more than 100 families with more than 550 participants. For 79 families, we were able to establish complete 3-generation pedigrees. Of those families, only 27 had more than 10 participating family members and 9 had more than 20 members participating in the study.

**Table 1:** Summary of families recruited

***Summary***	***Number***
Total numbers of samples collected	4200
Total number of families (pedigrees) where recruitment took place	103
Total number of participants affected by keloids in all families	550
Number of affected keloid participants recruited from families	321
Total number of families with complete three generation pedigrees	79
Number of families with greater than 20 participants	9
Number of families with greater than 10 participants	27

## Discussion

Scientific literature offers several recommendations on ethical, legal and cultural considerations for research in low resource settings and for recruitment of minority populations. Experiences with community engagement and informed consent for genetic studies in Africans [28] and for obtaining DNA samples from four populations including the Yorubas from Ibadan (Nigeria) have also been reported [29]. However, there is no report on issues that should be considered when recruiting entire families in rural and urban settings with genetic research as the main goal.

Successful recruitment of diverse populations such as the Yorubas for genetic studies is important for advancement in biomedical research. The Yoruba ethnic group was chosen for this linkage analysis approach because we were expecting that large families with keloids could be recruited. Due to the expected locus heterogeneity for causative variants leading to keloids [32-34], recruitment from the Yoruba population seemed ideal for finding significant linkage because of relatively low admixture [35,36] compared to many other populations.

To attain acceptance of a genetic study within multiple generations of a family, it is important to appreciate social, religious, and cultural beliefs that may impact participation. The Yoruba ethnic group that occupies mostly the western part of Nigeria highly values their cultural practices and religious beliefs. Although most Yorubas are Christians or Muslims, they retain their practice of worshiping different gods such as Sango (god of thunder), Obatala also known as Orisa-nla (the shaper, former, maker), Yemoja (god of the river) and Ogun (god of iron). All these beliefs create perceptions about the possible causes for a disease and often determine the approach to treatment. Traditional healers still have high influence on people’s decisions. Part of their health beliefs is that illnesses may originate from one or a combination of three forces; the magical practices (witches or sorcerers), natural events (environmental, hereditary) and supernatural occurrence (destiny or the individual’s double spirits) [37-39]. It has also been documented that many Nigerians approach treatment of diseases by utilizing Western medicine in combination with treatments offered by traditional healers and “spiritual” centers [37].

For example, one study reported that some Yorubas identified environmental factors such as diet and use of unorthodox traditional herbs as the cause for cancer [38]. The interest in experimenting with different treatments could explain why some of the participants in our keloid study reported treatment of their keloids with a combination of traditional herbs and Western medicine and why they were willing to contribute to science to find better treatment or cure. However, stigmatizing and/or painful diseases such as cleft lip and palate, cancer or keloids are by some perceived to be in part due to a “punishment” for evil deeds or result from retaliation by a jealous foe [37,38,40]. As a result, many Nigerians are weary of participating in studies that have the potential of “affecting” them or other family members, especially if they donate blood, saliva or hair samples. Recruitment was also affected by challenges that had already been described in other cultural environments [30,41]. Such issues included economic factors, lack of trust, different cultural background of researchers and study participants, or issues with time and transportation needed to reach remotely located family members.

Meeting regulatory and administrative requirements (IRB, ethics committees and FWA) as well as staff training was less of a problem compared to recruitment challenges. Research staff took the Collaborative Institutional Training Initiative (CITI) training for good clinical practice before they engaged in recruitment. An online education module for ethics training on research based on the Nigerian Code for Health Research Ethics has recently been developed and validated. Future studies will require research staff to take this module that encompasses the Nigerian cultural and social principles [42].

Our experience with this study confirmed the need to allocate extra time for all stages of the study. Extra time was needed to educate the public and prospective research participants about the study and to make up for delays due to travel logistics when recruiting relatives residing outside a proband’s home town or when family members were not available for recruitment in a timely manner. Additional challenges arose during the rainy season when treacherous road conditions led to extended delays. Unreliable public transport slowed down recruitment throughout the study period. While purchasing a car dedicated to the study might have been the most economical way to travel to recruitment sites, administrative barriers and uncertain liability issues prevented us from doing so.

Working with an institution with established research infrastructure versus a site without any research infrastructure or experience with foreign collaboration should be taken into consideration when planning a study as this will likely impact timely study initiation, staff recruitment, and research coordination. There is recognition that capacity building in African countries must be addressed for successful integration of human genetic studies [43]. Nigerians and specifically the Yorubas are generally not research-naïve. Participation of Yorubas or any underserved population in genetic studies can be enhanced by community involvement from inception of the study as well as by regular feedback [30], especially when the study design involves participation of family members from several generations. Our study plan included the assistance from probands or other individuals with keloids and contact with community leaders, kings or chiefs where applicable. Interaction with members of the oldest generation or children of old family members may be difficult when the culture of scientists and recruiters is different from that of the participants. To address these and other issues, our study involved research staff from the local community who were born and raised in the Yoruba culture. Visits to kings, chiefs, churches and mosques were useful to convince the community that blood and saliva samples would not be used for voodoo or juju. It was important and reassuring to be accompanied by research participants from their community at such meetings to demonstrate that nothing bad had happened to them since recruitment. It was also important to ensure that clinic or hospital administrators were aware of this study so that they could then encourage the population to participate. We were allowed to recruit in local palaces, churches and health centers/hospitals. Additionally, the research staff was available for recruiting during special holidays when relatives would return home for family celebrations. Cultural sensitivity during study coordination was ensured by a member of the US team that was born and raised in the Yoruba community.

Genetic analysis identified non-blood relatives in some families. Some pedigrees had to be updated several times as additional information became available. While scientists require accurate information about a participant’s position in a family it is more important for the traditional Yoruba family where individuals fit within a family than how they are biologically related. When obtaining family histories, we found that “adopted” distant relatives were sometimes identified as children or parents. We also encountered situations where friends of the family were reported as members of the family. These situations required that research staff approached a certain member of the family with clear questions to obtain accurate information before establishing a pedigree. During DNA analysis we still identified participants who did not fit in the family structure. The need for confidentiality in these situations was discussed among researchers and participants were either removed from the pedigree or in rare instances the family was eliminated from analysis. With increasing education, the community may become more aware of inheritance and biological relationships within a family and better understand the significance of genetic family studies. Until then, investigators should be prepared to address these factors in their study plan.

Even though remuneration is always a difficult topic for recruitment in low resource settings, it is still an incentive for participants who otherwise may not be able to travel to a recruitment site or miss some work/earning to volunteer for a study “without immediate benefit” to them. Other studies also reported that some participants expected more payment for their contribution [29], while others preferred to have free treatment/cure in lieu of money [44]. Investigators should be aware of the potential for abuse by opportunists that may impact recruitment. Our research protocol, for example, required that all study participants be treated and compensated equally.

In a review of Project SuGar, the author identified some dynamics that negatively affected recruitment of African-American families into genetic studies and offered suggestions to enhance recruitment [30]. Impacting factors included economic barriers, core values including cultural, spiritual and social factors, as well as health practices of the community. Previous studies have demonstrated that the majority of Nigerians would participate in research activities. Several approaches have been recommended or used to engage, recruit and retain Nigerians for genetic studies [45]. Such methods include the use of community advisory councils, incentives, local partners/champions, focus groups, awareness through public forums and less cumbersome wording during the informed consent process. Our study employed many of these suggestions.

Knowledge of genetic variations and their roles in disease pathology and for treatment outcome is significant in the quest to reduce health disparities. While there seems to be a recent increase in genetic studies conducted in African countries, the majority is usually in collaboration with and led by Western scientists. African countries must continue to be involved and take a leadership role in genetic studies so their people can benefit from the advances of the genomic and the post-genomic era. One could argue that the Yoruba people still have to offer a lot to science. Nigerians have been called upon to embrace the opportunity of improving their people’s quality of life with a timely response to technology gaps in genomics [46]. Initiatives such as the MalariaGen [47] and the Human Heredity and Health in Africa (H3Africa) project, among others, have begun to address this concern (H3Africa: Human heredity and Health in Africa, http://www.h3africa.org/).

## Conclusions

We believe that our recruitment experience amongst the Yorubas in Nigeria will be useful to other researchers who plan similar large genetic study studies in Africa or in other low resource settings. We have identified administrative and cultural differences as well as challenges due to insufficient infrastructure that can lead to delays in successful recruitment. As other genetic multigenerational studies are undertaken in low resource settings, investigators will recognize that a combination of methods that address the issues outlined in this article can enhance the success of their studies.

## Competing interests

The authors declare that they have no competing interests.

## Authors’ contributions

PBO, VO and EJR contributed to the conceptual development of the drafting of the manuscript. PBO, VO, SA, SOF, OMO and EJR contributed data for the manuscript and were involved in critical reading. All authors approved the final manuscript.

## Pre-publication history

The pre-publication history for this paper can be accessed here:

http://www.biomedcentral.com/1472-6939/15/65/prepub
